# The Clinical and Prognostic Implications of Pluripotent Stem Cell Markers Expression and Their Correlation with the WNT signal pathway in Hepatocellular Carcinoma

**DOI:** 10.31557/APJCP.2020.21.10.2961

**Published:** 2020-10

**Authors:** Nisreen AA Osman, Alzahraa Ibrahim Khalil, Rehab Kamal Yousef

**Affiliations:** *Department of Pathology, Faculty of Medicine, Minia University, Egypt. *

**Keywords:** HCC, SOX2, SOX9, p53, β-catenin

## Abstract

**Objectives:**

This study aimed to investigate the expression of SOX2, SOX9, p53, and β-catenin in hepatocellular carcinoma (HCC) and their correlation with clinicopathological parameters of prognostic importance.

**Materials and Methods:**

Seventy-five patients were enrolled in this study. All patients had full clinical and follow-up data and available paraffin blocks. Immunohistochemical analysis was performed and correlated with clinicopathological factors and patient survival.

**Results:**

We detected the positive expression of SOX2, SOX9, p53, and β-catenin in 76%, 50.7%, 50.7%, and 77.9% of HCC specimens respectively. All studied markers showed a significant increase in the expression in tumor tissue specimens compared to non-tumor tissue. Both SOX2 and SOX9 expressions were significantly associated with adverse prognostic factors in HCC. Significant positive correlations were found between SOX2 and SOX9 and both p53 and β-catenin expression (r= 0.528, 0.485 and; r = 0.253, 0.327, respectively; p< 0.0001 for both of them). Regarding survival, we found that HCC patients with positive SOX2 and SOX9 expressions had significantly shorter overall survival (p=0.0001, each). Additionally, larger tumor size, tumor grade, high stage, tumor multiplicity, presence of cirrhosis, tumor necrosis, high p53 expression, and positive β-catenin expression were independent predictors of worse survival. A multivariate Cox analysis revealed that tumor grade, stage, p53, and SOX2 expression were independent predictors of unfavorable prognosis in overall survival (p=0.0001, p=0.0001,p=0.033; and p=0.003, respectively).

**Conclusions:**

Our findings might provide an insight into SOX2 and SOX9’s role in HCC and suggest that SOX2 might be targeted for HCC therapy.

## Introduction

Hepatocellular carcinoma (HCC) ranks the fifth most common cancer in men, the seventh most common cancer in women, and the third leading cause of cancer-related mortality in the world (Ozakyol, 2017; Petruzziello, 2018) It is the most common primary hepatic malignancy, constituting around 90% of primary liver cancers (Elghazaly et al., 2018). 

In Egypt, HCC is a significant health burden. The incidence of HCC among all cancer types in Egypt is higher in males (33.63%) than in females (13.54%). The risk factors for HCC included hepatitis C and B infection, cirrhosis, metabolic syndromes, and alcohol abuse. Hepatitis C virus (HCV) is an epidemic that affected about 10-15% of people in Egypt (Elghazaly et al., 2018). Hepatocellular carcinoma related to chronic hepatitis C constitutes about 63% of cases and about 90% of them were of genotype 4 HCV infection (Yapali and Tozun, 2018). This leads to late discovery of HCC with subsequent poor prognosis (Elghazaly et al., 2018).

Multiple signaling pathways responsible for cellular proliferation, apoptosis, metabolism and cell cycle are established in HCC development. Several mutations in HCC are activated. They include mutations in the tumor suppressor gene p53, in CTNNB1Wnt/β-catenin pathway and others (Lee et al., 2014; Vilchez et al., 2016). 

The most commonly mutated gene in many human cancers is p53 being mutated in 50% of cancer cases with functional inactivation in 20% of cases. Not only p53 is a tumor suppressor protein but also p53 acts as a transcription factor (Levine and Oren, 2009). In HCC, up to 45% of hepatitis B virus (HBV) related HCC, >50% of aflatoxin B1-induced HCC and 13% of HCV-related HCC is associated with p53 mutation (Shiraha et al., 2013). Cancers exhibiting p53 mutations are reported to be more aggressive with resistance to chemotherapy (Mohamed et al., 2008; Aravalli et al., 2013).

β-catenin gene (CTNNB1), an oncogene encodes a β-catenin protein that plays a role in intercellular adhesion and involved in the development of HCC (Li et al., 2014; Wang et al., 2015). Notably, nucleocytoplasmic expression of β-catenin reflects a transcription factor function in the canonical Wnt/βcatenin signaling pathway, whereas membranous expression reveals its other function in cell-cell adhesion (Brembeck et al., 2006). Dysregulation of Wnt/β-catenin signaling is involved in early carcinogenesis and the aggressiveness of HCC. This implicates its role in cell survival, proliferation, and migration and invasion (Pez et al., 2013).

Despite the discovery of several pathways involved in hepatocarcinogenesis, still few of them are beneficial as targets for therapy and long term survival is still low. Therefore, there is a great demand for finding new targets involved in HCC progression. Sox (sex determining region Y) box gene superfamily, is incorporated in cell development, cell differentiation and embryonic stem cell properties. Also, it is considered as a transcriptional regulator (Lefebvre et al., 2007). Comparable to embryonic stem cells in organ development, cancer stem cells (CSCs) have the ability to initiate tumor development and resistance to both chemotherapy and radiotherapy (Dean et al., 2005; Phillips et al., 2006; O’Brien et al., 2007). So, the identification of CSC markers and their roles in cell signaling pathways might result in the construction of new therapeutic strategies. 

The transcription factor SRY (sex-determining region Y)-box 2 plays a key role in maintaining the stemness properties in cancer cells as it is involved in the reprogramming of differentiated cells into induced pluripotent stem cells (Yu et al., 2007; Yuan et al., 2010). SOX2 was associated with tumor progression of many tumors such as gastric (Li et al., 2004), pancreatic (Sanada et al., 2006), breast (Rodriguez-Pinilla et al., 2007), colorectal cancers (Han et al., 2012), and osteosarcomas (Basu-Roy et al., 2012). In HCC, SOX2 is involved in the progression of HCC and correlated with an aggressive phenotype (Huang et al., 2011). However little is known about the molecular role of SOX2 in HCC. 

The transcription factor-sex determining region Y-box 9-(Sox9), another member of Sox superfamily, plays a role in the organogenesis of several tissues. In hepatogenesis, Sox9 is expressed in the precursor cells during embryogenesis and during hepatocyte regeneration following liver injury (Furuyama et al., 2011; Yimlamai et al., 2014). Sox9 transcript level in HCC tissues is positively correlated with stemness markers including CD24, CK19, AFP, and EpCAM. Moreover, upon knockdown of Sox9 in HCC cells, downregulation of stemness-associated genes was detected. So that, Sox9 confers stemness through the Wnt/β-catenin signaling (Leung et al., 2016). Sox9 overexpression was associated with higher tumor grade, stage and poorer survival in HCC (Guo et al., 2012). 

The Wnt signal pathway and P53 have been proven as regulators of stem cells in many tissues (Luis et al., 2009; Li et al., 2012). P53 has been found to down-regulate multiple stem cell-associated genes, including SOX2, following DNA damage in embryonic stem cells (Li et al., 2012). SOX9 confers stemness through Wnt/β-catenin signaling (Leung et al., 2016). However, the correlations between cancer stem cells markers and Wnt signal pathway and P53 with clinicopathological features in cancers are still not elucidated.

In the present study, we aimed to investigate the expression of SOX2, SOX9 in HCC, and study their correlations with p53 and β-catenin. Also, to study their associations with clinicopathologic data and patient survival. 

## Materials and Methods

*Tissue samples*

This study consisted of 75 formalin-fixed, paraffin-embedded tissue specimens of hepatocellular carcinoma cases. They were chosen from the archives of the Pathology Department Minia, university Hospital during the period between 2009-2014. Forty-nine (49) cases were obtained by tru–cut biopsy and 26 cases were obtained by lobectomy. The pathology reports and hematoxylin and eosin (H&E) stained slides for all cases were reviewed to confirm the diagnosis. The histologic grade of HCC was done according to the 3-scale system; grade I, II and III (Schlageter et al., 2014). Tumors were staged according to the seventh edition of the tumor-node-metastasis (TNM) classification developed by the American Joint Committee on Cancer (AJCC) and International Union for Cancer Control (UICC) (Shindoh and Vauthey, 2014). Survival data was available for 3 years duration of patients’ follow up. The institutional ethics committees approving this research comply with the Declaration of Helsinki, 1975.

*Immunohistochemistry*

Four-micrometer tissue sections on positively charged slides were deparaffnized and rehydrated through xylene and graded ethanol solutions and then treated for 30 min with 3% hydrogen peroxide to block the endogenous peroxidase activity. For antigen retrieval, sections were treated with 0.1 mol/L citrate, pH 6.0, in a 700-W microwave oven for 20 min; ultra v block was added for slides of p53 immunostaining only for 5 minutes to block non-specific background staining. The sections were incubated overnight at 4°C in a humidity chamber with primary antibodies. Primary antibodies used were for p53 (monoclonal mouse antibody clone DO-7+BP53-12, ready to use, Lab Vision Laboratories), for β-catenin (polyclonal rabbit antibody, ready to use Lab Vision Laboratories), for SOX2 (Novus BiologicalsUSA, at dilution of 1:100 in PBS) and for SOX9 (rabbit polyclonal antibody, Santa Cruz Biotechnology, Inc. USA, at dilution of 1:50).

The reaction was detected with the avidin-biotin detection kit using diaminobenzidine (DAB) as the chromogen. Sections were counterstained with Mayer’s hematoxylin for 15 seconds then distalled water, after that dehydrated through graded ethanol alcohol, cleared in xylene and coverslipped before checked under the microscope. Negative control tissue sections were processed by omitting the primary antibody and slides incubated with PBS. Positive control was processed for each antibody (colonic carcinoma for p53, breast carcinoma for β-catenin, lung squamous cell carcinoma for SOX2 and basal layer of normal stratified squamous epithelium for SOX9).

*Immunohistochemical scoring*

Regarding p53, immunohistochemical expression was scored according to the percentage of nuclear positive tumor cells; the cutoff point was chosen as the median value of the average staining percentage which was 7%. Cases with a nuclear expression more than 7% are considered higher expression while cases with expression lower than 7% considered lower expression. For β-catenin, positive tumor cells were defined as cells with membranous and/or cytoplasmic staining. The median value of the average staining percentage was used as a cutoff point and it was 30% for both staining patterns. SOX2 immunostaining was scored according to the percentage of nuclear positive tumor cells; negative (if the percentage of stained tumor cells is ≤10) or positive (if percentage is>10 %) (Sun et al., 2013; Aboushousha et al., 2018). The cut-off value of positivity for SOX9 staining was set to 5% positive tumor cells/per 10 HPF (Richtig et al., 2017).

*Statistical analysis*

Chi-square and Fisher’s exact tests were used to compare categorical variables. Correlation between markers was evaluated using Spearman’s correlation coefficient. Results were considered statistically significant when the P value < 0.05. Data were analyzed using the Statistical Package for Social Sciences (SPSS) version 17 software.

*Survival analysis*

Kaplan-Meier survival curves and log-rank test statistics were employed to calculate three year overall patients’ survival and their differences. Multivariate regression analysis was carried out using Cox regression to assess the specific influence of each variable on survival in the presence of other variables. Only variables of significant value from the univariate analysis were entered into the Cox regression analysis. Hazard ratios (HRs) estimated from Cox models were reported as relative risks with corresponding 95% confidence intervals (CIs). A P value of <0.05 was considered significant.

## Results

*Clinicopathological parameters:*

The current study included 75 paraffin blocks from cases for patients suffering from hepatocellular carcinoma. The mean patients’ + age SD was 63.95 + 10.629 years and the median age was 63 years (ranged from 26-87 years). Most cases of male patients were in the age group less than the mean; 37 (62.7%) cases, while the majority of females were aged higher than the mean age; 12 (75%) and this difference was statistically significant (p<0.007). Hepatitis virus infection was found in 49 cases (65.3%). Hepatitis C virus infection was detected in 33 cases (67.7%) and hepatitis B virus infection was detected in 16 cases (32.7%). Collectively, there were more HCCs in male patients (59 cases, 78.7%); in cirrhotic livers (50 cases, 66.7%); with tumor grade 2 (38 cases, 50.7%) and with tumor stage III (27 cases, 36%). Fatty change was observed in non-tumor tissue in 8 cases (10.7%). Tumor tissue necrosis was found in 21 cases (28%). The details about clinicopathological data were presented in Table 1. 

*Immunohistochemical Results*

*Expression of studied markers in non-tumor tissue specimens*

Non-tumor tissue was found in 26 lobectomy cases and in 5 cases from tru-cut biopsies. Non-tumor tissue included areas of hepatitis or cirrhosis. High p53 expression was detected in hepatocytes adjacent to tumor tissue in 5/31 cases (16.1%). Positive β-catenin expression was found in the cytoplasm and nuclei of hepatocyte neighboring tumor areas in 8/31 cases (25.8 %). Both SOX2 and SOX9 positive staining was detected in 7/31 (22.6%) and 4/31(12.9%) of cases studied respectively. On comparing expression of the studied markers between non-tumor and tumor tissue specimens; significant results were obtained as mentioned in Table 2. 

*Immunohistochemical expression of studied markers in HCC cases*

*p53 expression*

**Figure 1:**
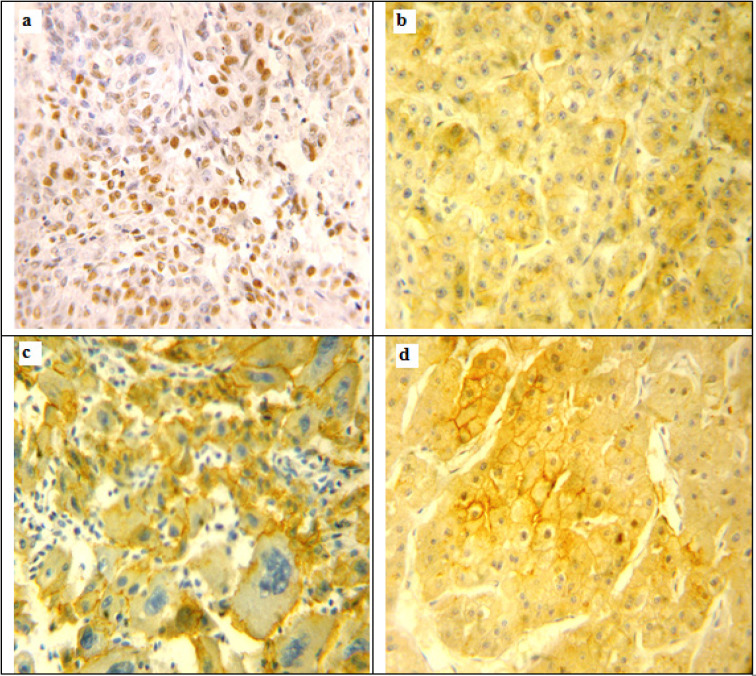
Immunohistochemical Expression of p53 and β-catenin in Hepatocellular Carcinoma Cases. a, High nuclear expression of p53 in hepatocellular carcinoma, grade II; b, Decreased membraneous and increased cytoplasmic expression of β-catenin in in hepatocellular carcinoma, grade II; c, Variable cytoplasmic or membraneous expression of β-catenin in hepatocellular carcinoma, grade III; d, Combined cytoplasmic, membraneous and nuclear expression of β-catenin in hepatocellular carcinoma, grade II. Original Magnification 200x, DAB was used as the chromogen and Haematoxylin as counterstain)

High p53 expression was found in 38/75 (50.7%) of HCC cases (Figure1a). High p53 expression was significantly more with the absence of fatty change and necrosis (p <0.028, <0.002 respectively). A statistically significant positive association was detected between high p53 expression and adverse prognostic factors of HCC including; larger tumor size, higher tumor grade and stage (p <0.048, =0.001, <0.006 respectively).Otherwise, no significant associations were found as seen in Table 3.

*β-catenin expression*

The incidence of abnormal β-catenin (decreased membraneous and/or increased cytoplasmic and nuclear expressions was variable in HCC studied cases (Figure 1b). Combined cytoplasmic and membraneous localization was found in 26 cases (30.2%); variable cytoplasmic or membraneous localization was found in 30 cases (34.9%, Fig.1c); while 19 cases (22.1%) lacked any β-catenin staining. Nuclear expression of β-catenin was observed in 6 cases; combined with cytoplasmic and membraneous expression, 4 of them were grade III and 2 of them were grade II (Fig.1d). As, the nuclear stain was detected in a low number of cases, they were not included in statistical analysis. A statistically significant positive association was detected between abnormal β-catenin expression and hepatitis infection, tumor size and tumor grade and stage (p <0.009, <0.008, <0.005 respectively) as shown in Table 4.

*SOX2 and SOX9 Expression*

**Figure 2:**
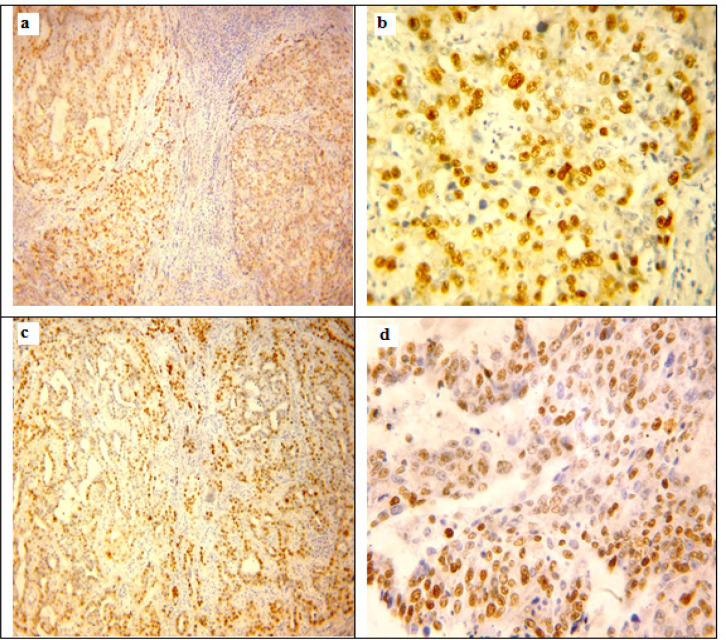
Immunohistochemical Expression of SOX2 and SOX9 in hepatocellular carcinoma cases. a, SOX2 positive expression in hepatocellular carcinoma, grade II with presence of cirrhosis and hepatitis; b, Nuclear SOX2 positive expression in hepatocellular carcinoma, grade II with scanty cytoplasmic staining; c, SOX9 positive expression in hepatocellular carcinoma, grade II with hepatitis; d, SOX9 positive expression in hepatocellular carcinoma, grade III. Original Magnification 100x for a, c and 200x for b, d, DAB was used as the chromogen and Haematoxylin as counterstain)

SOX2 positive expression was located mainly in the nucleus with scanty cytoplasmic staining (Figure 2a, b). We found 57of 75 (76%) HCC cases positive for that SOX2 expression. SOX2 positivity was significantly associated with younger age (p<0.037), male gender (p< 0.006) and presence of cirrhosis (p<0.0001). Regarding tumor characteristics, SOX2 positivity was significantly associated with larger tumor size (p<0.0001), with multiple tumors (p<0.0001), with the decreased amount of necrosis (p<0.002) and increasing tumor grade and stage of HCC (p<0.0001 for both) (detailed in Table 5).

Positive SOX9 expression was located mainly in the nuclei of neoplastic cells with accompanied cytoplasmic expression in 40 cases (Figure 2c, d). We found 38 of 75 (50.7%) HCC cases positive for that SOX9 expression. Negative SOX9 expression was observed in 10/75 (13.3%) cases and low SOX9 expression was found in 27/75 (36%) of HCC cases. SOX9 positivity was significantly associated with male gender (p< 0.021) and presence of cirrhosis (p<0.022). Regarding tumor characteristics, SOX2 positivity was significantly associated with larger tumor size (p<0.0001), with multiple tumors (p<0.0001), with the decreased amount of necrosis (p<0.025) and increasing tumor grade and stage of HCC (p<0.0001 for both) (detailed in Table 5).

*Relationship between expressions of studied markers in HCC cases*

The present work found that SOX2 and SOX9 expression rates were positively correlated with each other’s (Spearman’s rho correlation; rs= 0.766; p< 0.0001). A significant positive correlation was found between SOX2 and both p53 and β-catenin expression (Spearman’s rho correlation; rs= 0.528, 0.485 respectively; p< 0.0001 for both of them). Similarly, a significant positive correlation was found between SOX9 and both p53 and β-catenin expression (Spearman’s rho correlation; rs= 0.253, 0.327 respectively; p< 0.028, 0.004 respectively). A significant correlation was found between p53 and β-catenin expression rates (Spearman’s rho correlation; rs= 0.381; p< 0.0001). In HCC cases 38/75 (50.7%) were positive for both SOX2 and SOX9 expression (p= 0.001), 25/75 (33.3%) were negative for both of them and variable expression in 12/75 (16%) cases. 

*Correlation between expressions of studied markers and survival of patients*

The median overall survival period was 28 months (range 12-36 months) and the mean follow up period was 27.2 months. Forty patients (53.3 % of all cases studied) died during 3 years follow up. Using Kaplan- Meier method and log-rank test, there were significant associations between shorter overall survival of HCC and larger tumor size (p =0.0001), tumor grade (p =0.0001), high stage (p =0.0001, tumor multiplicity (p =0.0001), presence of cirrhosis (p =0.020), tumor necrosis (p =0.027), high p53 expression (p=0.0001), positive β-catenin expression (p=0.009), positive SOX2 expression (p=0.0001) and positive SOX9 expression (p=0.0001). On the other hand, no significant association was found between overall survival of HCC cases and either age (p =0.750), sex (p =0.080), presence of fatty change (p =0.685) or hepatitis virus infection (p =0.403).

In multivariate analysis, only tumor grade and stage among clinicopathological features have impact on overall survival (p=0.0001 for each). Regarding studied markers; p53 and SOX2 expression were independent predictor factors for overall survival (p=0.033; 0.003 respectively as shown in Table 6). 

**Table 1:** Clinicopathological Features of Patients with HCC (n= 75)

Clinicopathological features	Number (Percentage %)
Age (years)	
<64	41 (54.7)
>64	34 (45.3)
Sex	
Male	59 (78.7)
Female	16 (21.3)
Hepatitis	
Negative	26 (34.7)
Positive	49 (65.3)
Fatty change	
Absent	67 (89.3)
Present	8 (10.7)
Cirrhosis	
No	25 (33.3)
Yes	50 (66.7)
Tumor size (cm)	
<5 cm	30 (40)
>5 cm	45 (60)
Tumor Focality	
Solitary	36 (48)
Multifocal	39 (52)
Tumor necrosis	
Absent	54 (72)
Present	21 (28)
Tumor Grade	
I	20 (26.7)
II	38 (50.7)
III	17 (22.6)
Tumor Stage	
I	19 (25.3)
II	17 (22.7)
III	27 (36)
IV	12 (16)

**Table 2:** Comparison between Expression of Studied Markers in Tumor Tissue (Number 75) and Adjacent Non Tumor Tissue (number 31)

Marker	Non tumor tissue number (%)	Tumor tissue number (%)	P value
P53			
low	26 (83.9)	37 (49.3)	0.001*
high	5 (16.1)	38 (50.7)	
β catenin			
Negative	23 (74.2)	19 (25.3)	0.0001*
Positive	8 (25.8 )	56 (74.7)	
SOX2			
Negative	24 (77.4)	18 (24)	0.001*
Positive	7 (22.6)	57 (76)	
SOX9			
Negative	27 (87.1)	30 (40)	0.0001*
Positive	4 (12.9)	38 (50.7)	

Test of significance: Chi-square X^2^ and Fisher's exact tests. P-value < 0.05 is considered significant*

**Table 3:** Associations between Immunohistochemical Expression of p53 and Clinicopatholological Parameters in Hepatocellular Carcinomas Cases (75 Cases)

Parameter	P53 expression		P- value
	-ve/low expression (%)	High expression (%)	
Age (years)			
<64	18 (48.6)	23 (60.5)	0.302
>64	19 (51.4)	15 (39.5)	
Sex			
Male	31 (83.8)	28 (73.7)	0.286
Female	6 (16.2)	10 (26.3)	
Hepatitis			
Negative	10 (27)	16 (42.1)	0.17
Positive	27 (73)	22 (57.1)	
Fatty change			
Absent	30 (81.1)	37 (97.4)	0.028*
Present	7 (18.9)	1 (2.6)	
Cirrhosis			
No	16 (43.2)	9 (23.7)	0.072
Yes	21 (56.8)	29 (76.3)	
Tumor size (cm)			
<5 cm	19 (51.4)	11 (28.9)	0.048*
>5 cm	18 (48.6)	27 (71.1)	
Tumor Focality			
Solitary	21 (56.8)	15 (39.5)	0.134
Multifocal	16 (43.2)	23 (60.5)	
Tumor necrosis			
Absent	33 (89.2)	21 (55.3)	0.002*
Present	4 (10.8)	17 (44.7)	
Tumor Grade			
I	18 (48.6)	2 (5.3)	0.0001*
II	16 (43.2)	22 (57.9)	
III	3 (8.1)	14 (36.8)	
Tumor Stage			
I	16 (43.2)	3 (7.9)	0.006*
II	6 (16.2)	11 (28.9)	
III	11 (29.7)	16 (42.1)	
IV	4 (10.8)	8 (21.1)	

Test of significance: Chi-square X^2^ and Fisher's exact tests; P-value < 0.05 is considered significant*

**Table 4:** Associations between Immunohistochemical Combined Expression of β-catenin and Clinicopatholological Parameters in Hepatocellular Carcinomas Cases (75 Cases)

Parameter	Combined β-catenin expression			P- value
	-ve/-ve (%)	-ve/+ve or +ve/-ve (%)	+ve/+ve (%)	
Age (years)				
<64	12 (63.2)	16 (53.3)	13 (50)	0.669
>64	7 (36.8)	14 (46.7)	13 (50)	
Sex				
Male	12 (63.2)	25 (83.3)	22 (84.6)	0.16
Female	7 (36.8)	5 (16.7)	4 (15.4)	
Hepatitis				
Negative	5 (26.3)	6 (20)	15 (57.1)	0.009*
Positive	14 (73.7)	24 (80)	11 (42.3)	
Fatty change				
Absent	17 (89.5)	28 (93.3)	22 (84.6)	0.574
Present	2 (10.5)	2 (6.7)	4 (15.4)	
Cirrhosis				
No	8 (42.1)	8 (26.7)	9 (34.6)	0.528
Yes	11 (57.9)	22 (73.3)	17 (65.4)	
Tumor size (cm)				
<5 cm	13 (68.4)	11 (36.7)	6 (23.1)	0.008*
>5 cm	6 (31.6)	19 (63.3)	20 (76.9)	
Tumor Focality				
Solitary	12 (63.2)	15 (50)	6 (31.6)	0.294
Multifocal	7 (36.8)	15 (50)	13 (68.4)	
Tumor necrosis				
Absent	17 (89.5)	19 (63.3)	18 (69.2)	0.129
Present	2 (10.5)	11 (36.7)	8 (30.8)	
Tumor Grade				
I	10 (52.6)	8 (26.7)	2 (7.7)	0.005*
II	6 (31.6)	18 (60)	14 (53.8)	
III	3 (15.8)	4 (13.3)	10 (38.5)	
Tumor Stage				
I	8 (42.1)	9 (30)	2 (7.7)	0.074
II	5 (26.3)	5 (16.7)	7 (26.9)	
III	3 (15.8)	10 (33.3)	14 (53.8)	
IV	3 (15.8)	6 (20)	3 (11.5)	

Test of significance: Chi-square X^2^ and Fisher's exact tests. P-value < 0.05 is considered significant*; -ve/-ve: negative cytoplasmic and negative membraneous expression; -ve/+ve or +ve/-ve: negative cytoplasmic, positive membraneous expression or positive cytoplasmic, negative membraneous expression; +ve/+ve : positive cytoplasmic and positive membraneous expression.

**Table 5:** Associations between Immunohistochemical Expression of SOX2, SOX9 and Clinicopatholological Parameters in Hepatocellular Carcinomas Cases (75 Cases)

Parameter	SOX2 expression		P value	SOX9 expression		P value
	Negative (%)	Positive (%)		Negative (%)	Positive (%)	
Age (years)						
<64	6 (33.3)	35 (61.4)	0.037*	20 (54.1)	21 (55.3)	0.916
>64	12 (66.7)	22 (38.6)		17 (45.9)	17 (44.7)	
Sex						
Male	10 (55.6)	49 (86)	0.006*	25 (67.6)	34 (89.5)	0.021*
Female	8 (44.4)	8 (14)		12 (32.4)	4 (10.5)	
Hepatitis						
Negative	4 (22.2)	22 (38.6)	0.262	15 (40.5)	11 (28.9)	0.292
Positive	14 (77.8)	35 (61.4)		22 (59.5)	27 (71.1)	
Fatty change						
Absent	14 (77.8)	53 (93)	0.088	33 (89.2)	34 (89.5)	0.968
Present	4 (22.2)	4 (7)		4 (10.8)	4 (10.5)	
Cirrhosis						
No	14 (77.8)	11 (19.3)	0.0001*	17 (45.9)	8 (21.1)	0.022*
Yes	4 (22.2)	46 (80.7)		20 (54.1)	30 (78.9)	
Tumor size (cm)						
<5 cm	18 (100)	12 (21.1)	0.0001*	28 (75.7)	2 (5.3)	0.0001*
>5 cm	0 (0)	45 (78.9)		9 (24.3)	36 (94.7)	
Tumor Focality						
Solitary	18 (100)	18 (31.6)	0.0001*	32 (86.5)	4 (10.5)	0.0001*
Multifocal	0 (0)	39 (68.4)		5 (13.5)	34 (89.5)	
Tumor necrosis						
Absent	18 (100)	36 (63.2)	0.002*	31 (83.8)	23 (60.5)	0.025*
Present	0 (0)	21 (36.8)		6 (16.2)	15 (39.5)	
Tumor Grade						
I	18 (100)	2 (3.5)	0.0001*	20 (54.1)	0 (0)	0.0001*
II	0 (0)	38 (66.7)		14 (37.8)	24 (63.2)	
III	0 (0)	17 (29.8)		3 (8.1)	14 (36.8)	
Tumor Stage						
I	18 (100)	1 (1.8)	0.0001*	19 (51.4)	0 (0)	0.0001*
II	0 (0)	17 (29.8)		14 (37.8)	3 (7.9)	
III	0 (0)	27 (47.4)		4 (10.8)	23 (60.5)	
IV	0 (0)	12 (21.1)		0 (0)	12 (31.6)	

Test of significance: Chi-square X2 and Fisher's exact tests; P-value < 0.05 is considered significant*.

**Table 6:** Multivariate Analyses for Overall Survival in 75 Patients with HCC

Variables	B	SE	p-value	HR (95 CI)
Cirrhosis	0.193	0.347	0.579	1.212 (0.614-2.394)
Size	-0.231	0.595	0.698	0.794(0.247-2.548)
Tumor multiplicity	-0.312	0.555	0.575	0.732 (0.247-2.174)
Necrosis	-0.368	0.348	0.291	0.692 (0.350-1.370)
Grade	2.521	0.504	0.0001	12.445 (4.632-33.439)
Stage	1.461	0.408	0.0001	4.309 (1.937-9.588)
P53 expression	0.012	0.006	0.033	1.012 (1.001-1.023)
β-catenin expression	0.269	0.192	0.16	1.309 (0.899-1.906)
SOX 2 expression	-3.057	1.016	0.003	0.047 (0.006-0.345)
SOX 9 expression	0.263	0.461	0.568	1.301(0.527-3.208)

B, regression coefficient; SE, standard error; HR, hazard ratio; CI, confidence interval; p-value <0.05 is considered significant. Cox regression test is used.

## Discussion

Hepatocellular carcinoma is one of the most lethal malignancies. Despite the improvement in diagnosis and treatment modalities, the prognosis of HCC is still worse mainly due to high recurrence rate of HCC after resection (Fernandez-Sevilla et al., 2017). 

The aim of the present study was to investigate whether p53, β-catenin, SOX2 and SOX9 as a group of markers; act as transcription factors that are involved in hepatocarcinogenesis; could predict the malignant behavior of HCCs. In addition, we analyzed their prognostic value, their inter-correlation and their correlation to other histopathological parameters. Our positivity rates of the forementioned markers obtained by immunohistochemical assessment were in agreement with ranges reported in the literature (Guo et al., 2012; Sun et al., 2013; Liu et al., 2016; Richtig et al., 2017; Aboushousha et al., 2018; Khalaf et al., 2018). 

Many studies had focused on the activation of Wnt/β-catenin pathway and reported the involvement of various regulatory mechanisms that led to better understanding for their role in hepatocarcinogenesis. Upon comparing the expression of the studied markers in tumor and non tumor tissue, it was found that tumor tissue had significantly higher rates of expression. The same finding was reported in previous studies (Lee et al., 2014; Zhao et al., 2015; Richtig et al., 2017; Aboushousha et al., 2018; Khalaf et al., 2018). 

Many studies suggest that the presence of β-catenin mutation in HCC tumors is associated with prognosis and tumor behavior. Previous studies reported that the expression of β-catenin in the nucleus and cytoplasm may also be associated with high tumor grade, advanced stage and shorter overall survival (Lee et al., 2014; Lu et al., 2014; Zhao et al., 2015;Vilchez et al., 2016; Khalaf et al., 2018). The overexpression of p53 in this study is associated with larger tumor size, higher grade and stage and with shorter overall survival. Similar results were obtained in previous studies (Stroescu et al., 2008; Liu et al., 2016). The present study showed increased positivity of β-catenin and p53 expression in viral associated HCC than in non-viral HCC. The same result was reported previously (Liu et al., 2016; Khalaf et al., 2018). 

It was reported that SOX2 plays a pivotal role in oncogenesis and progression of multiple cancers including HCC (Sun et al., 2013; Zhao et al., 2015). In line with our results, It was found that immunohistochemical expression of SOX2 was more frequently over-expressed in HCC tissue compared to non-tumor tissue (Sun et al., 2013; Aboushousha et al., 2018). This result was confirmed by western blot, cell culture and qPCR analyses (Sun et al., 2013; Yin et al., 2013; Zhong et al., 2017). 

Our results showed that high expression of both SOX2 and SOX9 were significantly correlated with larger tumor size, tumor multiplicity, higher tumor grade, pathological stage and poor overall survival in univariate analysis. This was compatible with previous studies (Sun et al., 2013; Richtig et al., 2017; Aboushousha et al., 2018). 

In this study, we found that both the high expression of SOX2 and SOX9 were significantly associated with increased β-catenin expression and p53 expressioon. To our knowledge, this is the first study to assess the association between SOX2 and SOX9 and the Wnt signal pathway in HCC. Considering other tissues, a significant association was identified between the expression of Sox2 and β-catenin in cervical squamous cell carcinoma and lung carcinoma (Ji et al., 2014; Samulin Erdem et al., 2016; Guo et al., 2018). Additionally, we found that both the high expression of SOX2 and SOX9 were significantly associated with increased p53 expression. To our knowledge, this is the first study to the association between SOX2 and SOX9 and p53 in HCC. Considering other tissues, similar finding in NSCLC, SOX2 and TP53 expression were correlated (Samulin Erdem et al., 2016).

With regard to survival, we found that HCC patients with high p53 expression, high β-catenin expression, high SOX9 expression and low SOX2 expression had significantly worse survival time than those with low p53, β-catenin, and SOX9 and SOX2 expression by Kaplan–Meier analysis. Our findings suggested that p53, β-catenin, SOX9 and SOX2 possibly considered as a prognostic biomarkers in HCC. The same finding was reported by previous studies (Sun et al., 2013; Leung et al., 2016; Richtig et al., 2017; Zhang et al., 2019). 

On the level of multivariate analysis, only the high expression level of p53 and the low expression level SOX2 were associated with unfavorable prognosis in HCC. Taken together, p53 and SOX2 could be used as prognostic factors and be objected for molecular targeted therapy in HCC patients. An association between TP53 mutations and SOX2 copy number alterations was reported in lung cancer (Samulin Erdem et al., 2016). From the fore mentioned results we can conclude that the upregulation of Sox2 may play an important oncogenic role in HCC and can be considered as an acquired malignant proliferative phenotypic feature of tumor cells. 

More research is needed to determine the effect of molecular therapy targeting the stemness of Wnt/β-catenin pathway for treatment of HCC. Also, to investigate the possible regulation of P53 on SOX2 in several different HCC cell lines, as well as on molecular levels. 
